# The Impact of FDI on Urban PM_2.5_ Pollution in China: The Mediating Effect of Industrial Structure Transformation

**DOI:** 10.3390/ijerph18179107

**Published:** 2021-08-29

**Authors:** Taowu Pei, Lei Gao, Chao Yang, Chang Xu, Yu Tian, Weiming Song

**Affiliations:** 1 College of Economics and Management, China Agricultural University, Beijing 100083, China; peitaowu@bjfu.edu.cn; 2 School of Economics and Management, Yanshan University, Qinhuangdao 066004, China; 3 School of Economics and Management, Beijing Forestry University, Beijing 100083, China; yangchao99@163.com (C.Y.); or songwm@bjfu.edu.cn (W.S.); 4 Institute of Finance and Public Management, Anhui University of Finance and Economics, Bengbu 233030, China; 5 Institute of Ancient Books, Jilin University, Changchun 130012, China; yutian19@mails.jlu.edu.cn

**Keywords:** FDI, PM_2.5_, upgrading of industrial structure, rationalization of industrial structure

## Abstract

With the rapid growth of foreign direct investment (FDI), PM_2.5_ pollution in Chinese cities is increasing. Based on panel data for 271 Chinese cities from 2003 to 2016, this paper uses the dynamic spatial fixed-effects Durbin model to analyze the correlation between FDI and PM_2.5_ pollution and the effect of FDI on urban PM_2.5_ concentrations, as mediated by industrial structure transformation, which is clarified using Stata/SE 16.0. The results showed that PM_2.5_ pollution in China has significant spatial spillover effects, and the pollution haven hypothesis is applicable to Chinese cities. The industrial structure partially mediates the relationship between FDI and PM_2.5_. This paper proposes that local governments should promote the market-oriented reform of FDI to create a more convenient, legalized, and international environment for FDI and innovate the governance philosophy of only reducing the existing emissions. A top-level design and sound market supervision system of PM_2.5_ control are also needed.

## 1. Introduction

China has a stable political environment and huge consumer market and has hence become a hotspot of foreign direct investment (FDI). China’s Kearney FDI Confidence Index has ranked among the top 10 in the world for many years. FDI has also made an important contribution to the rapid development of China’s economy, by making up for shortages in capital and providing management experience and advanced production technologies [1,2]. According to data from the Ministry of Commerce of China, China’s FDI increased from USD 1.2 billion in 1979 to USD 138 billion in 2018 [3]. However, while China’s economy has achieved rapid development through FDI, there have been serious conflicts between economic development and environmental protection, which can undermine the sustainability of economic growth [4,5,6,7,8].

**Figure 1:**
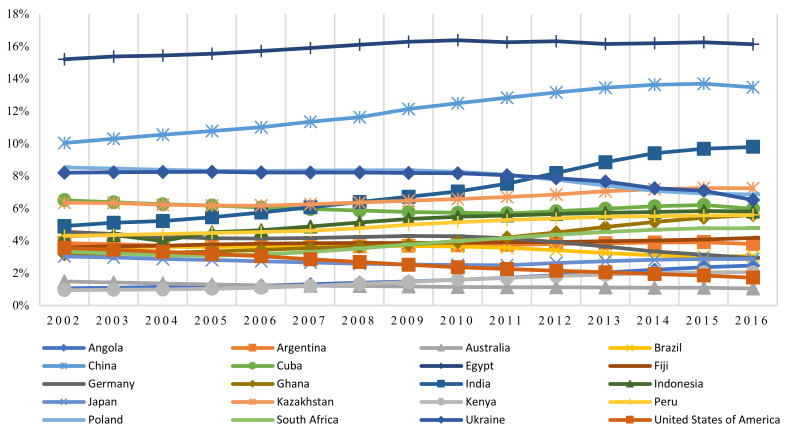
Percentage of total deaths caused by ambient particulate matter pollution in various countries from 2002 to 2016.

**Figure 2:**
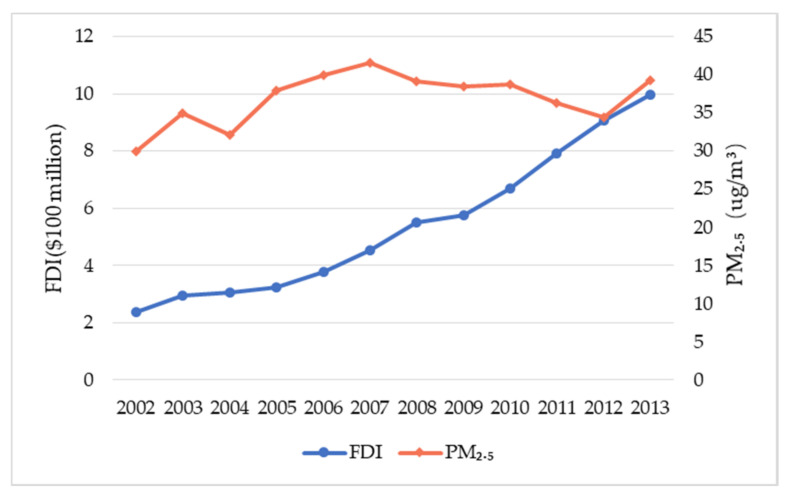
Average FDI and PM_2.5_ of 271 cities in China from 2002 to 2013.

Among many environmental problems in China, PM_2.5_ pollution has been of significant concern in recent years. Disasters in Meuse Valley, Donora, and London have acted as a warning to China that economic development should not be at the cost of human health and the environment [9,10,11]. According to data from the Global Burden of Disease study of 2017, air pollution ranked fourth among the factors promoting death and disability in China, contributing an additional 1.7% compared with 2007 [12]. Figure 1 shows the percentage of total deaths caused by ambient particulate matter pollution in a number of countries from 2002 to 2016, and indicates that in recent years, this figure in China is the second highest in the world, behind only Egypt. Thus, PM_2.5_ pollution in China poses a significant threat to human health. Figure 2 shows the average FDI and PM_2.5_ of 271 cities in China from 2002 to 2013 and indicates a similar upward trend in all of them. The question of whether the rapid growth in FDI has led to an increase in urban PM_2.5_ pollution should be addressed.

We agree with Grossman and Krueger (1995) that the impact of FDI on the environment is affected by economic scale, industrial structure, and production technology [13]. However, in the existing literature, results based on this framework differ widely, including among similar areas and time periods [14,15]. We believe that this is mainly due to a lack of comprehensive consideration of structural effects. Most studies pay more attention to the impact of FDI on the service-oriented processes of industrial structures, ignoring that FDI also affects the degree of coupling between production factors and the economic output of the host country [16]. Specifically, they only focus on the impact of upgrading the industrial structure and ignore that of industrial structure rationalization. Rational industrial structure can promote allocation efficiency in terms of production resources, to the benefit of both the environment and the economy [17,18,19]. A lack of consideration of industrial structure rationalization factors may lead to biases in the measurement of structural effect, which will in turn affect comparisons between scale and structure effects and technological effect, and thus determination of whether a pollution haven or halo has been created. Based on this, this paper incorporates industrial structure rationalization factors into its framework and explores the mediating effect of industrial structure on the relationship between FDI and PM_2.5_.

Based on panel data for 271 Chinese cities from 2003 to 2016, this paper uses the dynamic spatial econometric model to explore the impact of FDI on urban PM_2.5_ pollution in China through two paths: upgrading and rationalization of the industrial structure. This paper has three novel aspects. (1) At present, work on the impact of FDI on PM_2.5_ at the city level is relatively rare. Based on data for prefecture-level cities, this paper uses a dynamic spatial econometric model to measure the spatio-temporal effect of FDI on PM_2.5_ in more detail. (2) The mediating effect of industrial structure on the relationship between FDI and PM_2.5_, which has rarely been studied in depth previously, is analyzed. (3) Industrial structure rationalization factors are incorporated into our framework, unlike in existing literature, and the impact of industrial structure transformation on urban PM_2.5_ is assessed from two perspectives.

The remainder of this paper is organized as follows: Section 2 includes a review of previous work from relevant theoretical and empirical perspectives. Section 3 discusses the model specification and data sources, Section 4 provides empirical results, Section 5 provides the discussion, and Section 6 provides conclusions and discusses policy implications.

## 2. Literature Review

### 2.1. FDI and Environment

Whether FDI exerts undue environmental pressure on the host country has long been controversial. The famous pollution haven hypothesis (PHH) was first proposed by Copeland and Taylor (1994) when they studied the relationship between north-south trade and the environment, which holds that under the conditions of an open economy, the free trade will lead to the continuous transfer of polluting production practices or enterprises from developed countries to developing countries [20]. This is because developed countries generally have high environmental awareness, and they usually implement relatively strict environmental regulations, which will undoubtedly promote the cost of polluting industries in developed countries. Therefore, compared with other competitors, manufacturers have a significant cost advantage in countries with lower environmental standards. For this reason, the dirty industries of developed countries will naturally shift to developing countries. The latter will become a pollution haven [8,20,21,22,23,24,25].

However, the pollution halo hypothesis holds that cleaner production technologies and management experience will be transferred along with FDI. Through the demonstration effect and learning-by-doing, the environmental quality of host countries will be improved through FDI, according to this latter hypothesis [26,27,28,29]. It is not clear which hypothesis is most applicable to urban PM_2.5_ pollution in China.

### 2.2. FDI and Industrial Structure

Chenery and Strout (1966) propose the theory of two gaps and believe that introducing foreign capital can offset the dual gaps in the savings and foreign exchange reserves of developing countries and adjust the industrial structure through capital allocation to achieve economic growth and industrial structure optimization and upgrading [30]. Jensen (2002) takes Poland as a sample and finds that FDI is biased toward Polish technology-based industries. The export value of Polish technology industries has increased with FDI [31]. Through the analysis of Czech, Kippenberg (2005) finds that FDI promotes the further optimization of the industrial structure of Czech, and through demonstration effect and competition effect, it has become the driving force to promote the development of the industrial economy of Czech [32]. Nefussi and Schwellnus (2010) also find that a large number of FDI flows into high-tech industry, thus promoting the adjustment and upgrading of industrial structure in France [33].

In addition, scholars believe that FDI has no significant impact on the industrial structure of the host country. Hunya (2002) uses the export data of Romania and finds that FDI in Romania tends to labor-intensive industries. Therefore, FDI does not change the traditional industrial structure, but further solidifies the industrial structure of Romania [34]. Demir and Duan (2018) studied FDI in 108 host countries and 240 home countries from 1990 to 2012 and found that FDI has no significant impact on productivity of various industries in each country [35].

It can be seen that there is no consensus on the impact of FDI on the industrial structure of the host country. The above studies pay more attention to industrial upgrading of the host country, while the study on its rationalization of industrial structure is scarce.

### 2.3. Industrial Structure and Environment

Ahluwalia et al. (1979) argue that the transformation of industrial structure is a key factor to understand the difference between developed and developing economies. Especially in developing economies, it may affect the transfer and allocation of resources among different industries and then cause the difference in pollution emissions of different industries [36]. Dinda and Coondoo (2000) also believe that the transformation of industrial structure to knowledge-technology-intensive tertiary industry is conducive to the reduction of pollution and environmental improvement [37].

Grossman and Krueger (1995) believe that the industrialization of East Asian countries can be roughly divided into three stages: light industry, heavy chemical industry, and electronic industry. The different stages respectively correspond to the national economic system dominated by labor-intensive, resource-intensive, and technology-intensive industries. It is found that there is an inverted U-shaped relationship between environmental pollution and industrialization process. Thus, they divide the environmental impact of FDI into three parts: economic scale, industrial structure, and production technology [13]. We have combed and summarized the literature based on this framework in Table 1. It will help us see the differences between these studies more simply and clearly.

## 3. Methodology

### 3.1. Model

We used the STIRPAT model as our theoretical and analytical framework, which has been widely used by scholars [52,53]. Ehrlich and Holdren (1971) first proposed a model to describe the influence of population, affluence, and technology (IPAT) on environmental impact [54]. However, the simplicity of the IPAT model does not account for the numerous other factors related to human impact on the environment. To fill this gap, Dietz and Rosa (1997) proposed a stochastic version of IPAT called STIRPAT, which provides a relatively quantitative framework for analyzing environmental impacts [55]. The basic form of the STIRPAT model is:

(1) $$I_{it}=\alpha P_{it}^{b}A_{it}^{c}T_{it}^{d}e_{it}$$

where I represents environmental impact; P, A and T represent population, affluence, and technology respectively; a, b, c and d represent the coefficients of the explanatory variables to be estimated; $e_{it}$ stands for random error; the subscript i is the sample individual, which refers in this research to the province; and t is the sample time, which in this research is the year.

Researchers have expanded the STIRPAT model and incorporated new variables. For example, York et al. (2003) added the quadratic term of population [52], Andreoni and Levinson (2001) added the quadratic term of per capita output [56], and Martínez-Zarzoso and Maruotti (2011) added the urban population proportion [57]. We used an extended version of the STIRPAT model to test the relationship between FDI and PM_2.5_. In this model, referring to the practice of Wang et al. (2016), all variables are converted to the natural logarithmic form [58]. Our expanded version of the model is as follows:

(2) $$\ln{\mathrm{PM}}_{2.5it}=\beta_{1}\ln P_{it}+\beta_{2}\ln A_{it}+\beta_{3}\ln T_{it}+\beta_{4}\ln{\mathrm{FDI}}_{it}+\beta_{5}\ln{\mathrm{US}}_{it}+\beta_{6}\ln{\mathrm{RS}}_{it}\;+\beta \ln X_{it}+\alpha_{i}+\gamma_{t}+\varepsilon_{it}$$

The explained variable is the degree of environmental pollution, and we used PM_2.5_ as the measurement index. On the left side of Equation (2), ${\mathrm{PM}}_{2.5}{}_{it}$ represents the PM_2.5_ pollution of city i in year t.

The right side of Equation (2) contains the explanatory variables, where: 

P represents population, which is measured by population density. The calculation method is the total population of city i in the year t, divided by the administrative area. There are differences in the population and administrative areas of each city. Simply using the population size cannot scientifically account for the impact of population on PM_2.5_; therefore, we use the number of people per unit area to measure the relationship between population concentration and PM_2.5_ pollution. 

A represents the degree of affluence, which is measured by GDP. The larger the value, the greater the wealth of the city. GDP is based on 2002 data adjusted using the GDP deflator, which is actual GDP excluding price changes. 

T represents technology, which is measured by energy efficiency. The calculation method is the actual GDP of city i in the year t, divided by the electricity consumed by the city in that year. Technological innovation is important for controlling air pollution, and energy efficiency improvements are driving forces for perfecting environmental technologies [59,60]. Therefore, we measure energy efficiency through GDP created by each unit of electricity. The larger the value, the higher the output level of the same energy consumption, that is, the higher the energy efficiency.

FDI is the explanatory variable, which is measured by the total amount of registered investment by foreign enterprises in city i in the year t. It is converted from USD into RMB based on the average exchange rate and GDP deflator published by the National Bureau of Statistics of China and taking 2002 as the base period.

US is the industrial structure upgrading index. The upgrading of the industrial structure of the host country indicates the development process of industrial structure, which develops from a lower-level state to a higher one, that is, it is a constantly improving and upgrading process of overall level of industry. This index can reflect whether the industrial structure is developing in the direction of “service-oriented”. When the value of upgrading is on the rise, it indicates that the economic structure is currently developing toward service-oriented and that the industrial structure is upgrading. Using the method proposed by Gan et al. (2011), the index is calculated as the ratio of the output value of the tertiary industry (According to the industry classification standard of China “*Classification of National Economic Industries* (GB/T 4754-2011)”: the primary industry refers to agriculture, forestry, husbandry, and fishery (excluding service industries of agriculture, forestry, husbandry, and fishery). The secondary industry refers to mining (excluding mining auxiliary activities), manufacturing (excluding the repair of metal products, machinery and equipment), electricity, heat, gas and water production and supply, and construction. The tertiary industry is the service industry, which refers to industries other than the primary industry and the secondary industry.) to the output value of the secondary industry [7,16,49].

RS is the industrial structure rationalization index. Many studies use the method proposed by Gan et al. (2011) to construct this index [16,48,61]. However, this method ignores the role of absolute values. Discrepancies among industries may result in “false rationalization” of the industrial structure [62]. Therefore, this paper modifies the method proposed by Jia et al. (2016) [63]. The calculation formula is as follows:

(3) $$\mathrm{RS}=\sum_{i=1}^{n}\frac{Y_{i}}{Y}\times \frac{Y_{i}/L_{i}}{Y/L}=\sum_{i=1}^{n}\frac{Y_{i}}{Y}\times \frac{Y_{i}/Y}{L_{i}/L}$$

where *Y* is the output value, *L* is the labor force, *i* represents the *i*th industry, and *n* is the number of industrial sectors. When $Y_{i}/L_{i}=Y/L$, RS=1, the economy has reached a balanced state.

*X* is the control variable group, including urbanization, park green space area, and total bus and tram traffic volume. Local governments may sacrifice the environment during the urbanization. We draw on the practice of Shao et al. (2019) and use the stable light data to measure the degree of urbanization of a city [47]. Park green space is the “green lung” of cities in which plants play a significant role in reducing ambient particulate matter pollution and improving air quality [64]. In addition, traffic is one of the important sources of PM_2.5_ pollution. Due to a lack of private car data at the prefecture level, this paper uses the total bus and tram passenger volume to measure the traffic intensity of cities [65,66].

$\alpha_{i}$ represents the fixed effect in city i and is used to control the characteristics that do not change over time. $\gamma_{t}$ refers to yearly fixed effect, which is used to control the time-varying omitted variables and stochastic shocks that are common to all cities.

Due to the strong spatial spillover effect of the dependent variable (Appendix A for the test of spatial effect), PM_2.5_, we need to consider spatial factors in the model and adopt a spatial econometric model [67]. If the spatial effect is ignored in the econometric model, the estimation result will be biased [66,68,69]. According to Formula (4), the model is as follows:

(4) $$\ln{\mathrm{PM}}_{2.5it}=\rho \overset{N}{\underset{j=1}{\sum }}w_{ij}\ln{\mathrm{PM}}_{2.5jt}+\beta_{1}\ln P_{it}+\beta_{2}\ln A_{it}+\beta_{3}\ln T_{it}+\beta_{4}\ln{\mathrm{FDI}}_{it}\;+\beta_{5}\ln{\mathrm{US}}_{it}+\beta_{6}\ln{\mathrm{RS}}_{it}+\beta \ln X_{it}+\alpha_{i}+\gamma_{t}+\varepsilon_{it}$$

where *w* is the spatial weight matrix, and common spatial weight matrices may be a 0–1 matrix, an inverse distance matrix, an economic geography matrix, etc. To better investigate the spatial correlation characteristics of PM_2.5_ and consider the endogenous problem of the economic distance matrix, this paper uses the inverse distance matrix as the spatial weight matrix. *ρ* is the spatial factor of the dependent variable.

Furthermore, Equation (4) implicitly assumes that PM_2.5_ will change instantaneously with a change in local factors, that is, there is no adjustable time lag effect. However, in reality, environmental pollution often has path-dependent characteristics in which the previous value has a significant impact on the current one [70]. Therefore, on the basis of Equation (4), we add a time lag factor for PM_2.5_ in Equation (5). Here, *τ* is the dynamic factor of the dependent variable.

(5) $$\ln{\mathrm{PM}}_{2.5it}=\tau \ln{\mathrm{PM}}_{2.5it-1}+\rho \overset{N}{\underset{j=1}{\sum }}w_{ij}\ln{\mathrm{PM}}_{2.5jt}+\beta_{1}\ln P_{it}+\beta_{2}\ln A_{it}+\beta_{3}\ln T_{it}\;+\beta_{4}\ln{\mathrm{FDI}}_{it}+\beta_{5}\ln{\mathrm{US}}_{it}+\beta_{6}\ln{\mathrm{RS}}_{it}+\beta \ln X_{it}+\alpha_{i}+\gamma_{t}+\varepsilon_{it}$$

Equation (5) is the dynamic spatial panel model adopted in this paper. The dynamic spatial panel model can comprehensively reflect the time lag and spatial lag effects of PM_2.5_; thus, it is helpful for obtaining more robust estimation results.

### 3.2. Data Sources

The PM_2.5_ concentration data were obtained from the Center for Social and Economic Data and Applications at Columbia University [71,72] and the data of stable light published by the National Oceanic and Atmospheric Administration [73,74]. The data for remaining independent variables in this paper were derived from the China City Statistical Yearbooks [75]. Based on availability, we used data for 271 cities in the period 2003–2016 to establish a complete panel data set. Table 2 shows the descriptive statistics of variables.

The data used in this paper is short panel data; thus, we employed the approach of Harris and Tzavalis (1999) to test unit root [76]. Table 3 shows the results of HT test. In the case of level values, there are three variables that are nonstationary. After the first-order difference of all variables, all the data series are stationary.

Then, we took a cointegration test for all variables. Due to involving many independent variables, we employed the approach of Kao (1999) to test cointegration [77]. In Table 4, the results of the cointegration test significantly reject the null hypothesis, indicating that there is a stable long-term equilibrium relationship between the independent variables and control variables and dependent variable. The original data can be used for regression, and the model setting is applicable.

## 4. Empirical Results

### 4.1. Verification of the PHH

Due to the model setting, we took the spatial autoregressive models (SAR) and spatial Durbin models (SDM) as alternative models and analyzed them by using Stata/SE 16.0. The results of the Wald and Lratio tests showed that the independent variables have significant spatial effects; thus, the null hypothesis cannot be rejected. This indicates that the SDM cannot be simplified into the SAR. The results of the Hausman test significantly rejected the null hypothesis; thus, this paper will adopt the fixed-effect SDM as the main analysis model. In addition, limited by space, we will analyze and discuss the empirical results in more detail in Section 4.

In Table 5, column (1) shows the results of static fixed-effect SDM, and columns (2)–(5) show the results of dynamic fixed-effect SDM, accounting for the temporal lag effect of dependent variables. The Akaike information criterion (AIC) and Bayesian information criterion (BIC) were used for model selection [78,79]. We found that the AIC and BIC index of model (4) is smaller than others. Thus, we will focus on the results in column (4) below with other results listed for reference.

In the results shown in column (4) of Table 5, the spatial lag coefficient of PM_2.5_ is significantly positive, which also demonstrates that urban PM_2.5_ pollution in China has clear spatial agglomeration. This is due to the dual effects of atmospheric movement and economic activities. The temporal lag coefficient of PM_2.5_ is also positive (significance level of 1%). For each 1% increase in PM_2.5_ pollution in the current period, it will increase by about 0.14% in the next period. This indicates that the change in PM_2.5_ is clearly path-dependent. If the concentration of PM_2.5_ is high in the current period, it will likely continue to rise in the next period.

In terms of FDI, the regression results of Table 5 show that FDI has a positive impact on PM_2.5_ pollution in Chinese cities. This shows that the introduction of FDI is not conducive to alleviation of PM_2.5_ pollution in Chinese cities, and the PHH is therefore applicable in China. Leal et al. argue that it is predictable in the developing economies, due to certain evident factors, such as comparative advantages on labor factor or energy [80].

As shown in column (4) of Table 5, the impact of the industrial structure upgrading index on PM_2.5_ is positive (significance level of 1%); if the degree of industrial structure upgrading increases by 1%, the PM_2.5_ concentration will be increased by 0.12%. The impact of the industrial structure rationalization index on PM_2.5_ is positive (significance level of 1%); if the degree of industrial structure rationalization increases by 1%, the PM_2.5_ concentration will be reduced by 0.13%. Regarding the other control variables, PM_2.5_ pollution shows a significant “U” curve trend with economic growth. Energy efficiency has negative effects on PM_2.5_, while urbanization, population density, and traffic intensity will exacerbate PM_2.5_ pollution [81].

### 4.2. Decomposition of Direct and Indirect Effects

When variables have a spatial spillover effect, they not only affect the PM_2.5_ concentration in the local city, but also of that in neighboring cities. According to Lesage and Pace, the effect of variables on PM_2.5_ pollution can be decomposed into direct effects and indirect effects [82]. This paper uses a dynamic model, which can be similarly decomposed into short- and long-term effects [41,49].

Table 6 shows the decomposition results of the model in column (4) of Table 5, which shows significantly in the long term at the level of 1%, but not in the short term. This indicates that the regulation of PM_2.5_ in Chinese cities is a protracted struggle. It is necessary to beware of the effect of boiling frog.

In addition, the introduction of FDI is not conducive to improvement of PM_2.5_ pollution in a given city and the PHH is applicable in the long run. However, the indirect effect of FDI is negative, which indicates that the introduction of local FDI will effectively reduce the PM_2.5_ concentration of neighboring cities in the long run.

The coefficient of the direct effect of the industrial structure upgrading index is negative. This shows that the optimization of a city’s industrial structure is a long-run effect policy. The development of the producer service industry may increase local environmental pressure, but only after the throes of the adjustment; the optimization of industrial structure will have the cleaning feature.

The coefficient of the direct effect of the industrial structure rationalization index is positive, indicating that the unreasonable industrial structure will continue to cause inefficient resource utilization of Chinese cities, resulting in wasted resources and urban pollution.

Regarding the other control variables, economic growth, urbanization, population density, and traffic intensity will exacerbate PM_2.5_ pollution in the long run. And the park green space area of city will significantly alleviate PM_2.5_ pollution.

### 4.3. Robustness Test

#### 4.3.1. Replace Core Independent Variable

The estimated results of the industrial structure upgrading index in Table 5 are inconsistent with expectations. In order to further determine whether the impact of the industrial structure upgrading index on PM_2.5_ is stable, it is necessary to conduct a robustness test. We replaced the previous calculation with the proportion of GDP of the tertiary industry. Column (1) in Table 7 is the estimated result after variable replacement. The result shows that the degree of industrial structure upgrading still has a positive impact on Chinese urban PM_2.5_ pollution. Compared with the results of column (4) in Table 5, the estimation result of the industrial structure upgrading index is robust.

#### 4.3.2. Consider the Effect of Capital Accumulation

In the research on economic growth, it is important to consider the effect of capital accumulation and learning-by-doing [83]. As a form of capital, FDI also has an accumulation effect. Therefore, we considered the FDI’s first-order lag variable L.lnfdi in the control variables. Column (2) in Table 7 is the estimated result that considers the accumulation effect of FDI. Compared with the results of the column (4) in Table 5, the estimation results of other variables are less different; thus, the previous results are still robust.

### 4.4. Testing the Mediating Effects

The empirical results in Table 5, Table 6 and Table 7 show that the PHH is applicable to Chinese cities, and there is significant path dependence. Using the method proposed by Baron and Kenny, we explored the mediating effect of industrial structure transformation on the relationship between FDI and PM_2.5_ [84].

#### 4.4.1. Mediating Effect of the Industrial Structure Upgrading Index

The estimated results in Table 5 and Table 6 show that the upgrading transformation of urban industrial structure cannot show the cleaning features in the short run. The upgrading industrial structure index will have a positive impact on the PM_2.5_ concentration of Chinese cities, and the PHH indicates that the introduction of FDI will reduce the industrial structure upgrading index, which is established. Therefore, considering the policy implications of the mediating effect, we believe that there is no room for policy adjustment in the short run. Whether the policy supports the service-oriented transformation or not, it is not conducive to reducing the city’s PM_2.5_ concentration. The estimated results in Table 6 show that, in the long run, the industrial structure upgrading will have a negative impact on the Chinese urban PM_2.5_ pollution. Therefore, it is more practical to test the mediating effect based on the estimated results of the long run effect. Thus, we decided to explore whether the path of “FDI-industrial structure upgrading-PM_2.5_” is established based on the long run direct effects in Table 6.

Table 8 shows the mediating effect of the industrial structure upgrading index. As shown in the table, the coefficients of FDI in all models are significant at the 1% level and the coefficient of the industrial structure upgrading index in column (3) are significant at the 1% level. Thus, we can draw a conclusion that the industrial structure upgrading index has a partial mediating effect on the relationship between FDI and PM_2.5_. This shows that the path of “FDI-industrial structure upgrading-PM_2.5_” is established. FDI has increased PM_2.5_ pollution by reducing the degree of Chinese urban industrial structure upgrading. The mediating effect of the industrial structure upgrading index accounts for 10.29% of the total effect of FDI.

#### 4.4.2. Mediating Effect of the Industrial Structure Rationalization Index

In the previous analysis, the degree of urban industrial structure rationalization played a key role in whether FDI spillover effect can be exerted, including advanced emission reduction technology and management experience. A higher degree of the industrial structure rationalization indicates more effective factor allocation and resource utilization. The combined effect of demonstration and learning-by-doing will help alleviate environmental pollution in Chinese cities. Thus, we will take the industrial structure rationalization index as the mediating variable, and further explore whether the path of “FDI-industrial structure rationalization-PM_2.5_” is established based on the estimated results in Table 5.

Table 9 shows the mediating effect of the industrial structure rationalization index. As shown in the table, the coefficients of FDI in all models are significant at the 10% level and the coefficient of the industrial structure rationalization index in column (3) is significant at the 1% level, indicating that the industrial structure rationalization index partially mediates the relationship between FDI and PM_2.5_. The path of “FDI-industrial structure rationalization-PM_2.5_” is established. FDI has increased PM_2.5_ pollution by reducing the degree of Chinese urban industrial structure rationalization. The mediating effect of the industrial structure rationalization index accounts for 49.94% of the total effect of FDI. Compared with the industrial structure upgrading index, industrial structure rationalization index has a stronger mediating effect.

## 5. Discussion

### 5.1. Impact of FDI on Urban PM_2.5_ in China

The regression results in Table 5 and Table 6 show that PHH is applicable based on urban PM_2.5_ pollution in China, and PM_2.5_ has a significant spatial spillover effect. The possible reasons for this are as follows:

First, FDI helps to expand the scale of economic activity and promotes economic growth of a city. If there is no transfer of polluting industries, and the original economic industrial structure is maintained, the larger economic scale leads to more resource input and consumption. Leal et al. (2021) argues that in the developing economies, the stock of FDI increases sustainable development through inward FDI, however, it increases environmental degradation as well [80]. This view is similar to that of Tang et al. (2016) [85]. Onafowera and Owoye (2014) also believe that there is a positive long run relationship between economic growth and pollution [86].

In addition, in the early stages of China’s economic development, several cities introduced FDI to create jobs and improve the local economy at the expense of the environment [24]. Furthermore, as mentioned by Shao et al. (2016) and Cheng et al. (2020), GDP-oriented performance appraisal will cause blind competition among local governments for FDI that ignores green issues [8,41]. Taguchi and Murofushi (2010) also argues that ineffective control of air pollution may cause a pollution haven effect through the interjurisdictional competition for polluted industries on regional latecomers [87]. However, according to the results in the Table 6, the adsorption effect of FDI on labors may reduce energy consumption and PM_2.5_ pollution in neighboring cities.

Lastly, many studies have shown that reasonable labor allocation would assist in maximizing the technological effect of FDI [17,19]. In particular, to localize management experience, more advanced labors are needed for domestic enterprises. Therefore, it is not certain whether the demonstration effect of foreign enterprises is applicable.

According to the empirical results presented herein, the scale and structure effects of FDI in China dominate over the technical effect, which is not fully exerted. Thus, the PHH is applicable in China.

### 5.2. The Throes of Industrial Structure Upgrading Transformation

We assumed that the tertiary industry is a clean industry, which has a low contribution to air pollution in the beginning. But the empirical results in Table 5 are the opposite, which is similar with Zhang et al. (2020) [7]. We believe that producer services are main source, especially transportation. The sum of the fuel consumption of China’s public transportation and private vehicles accounted for 92.70% of total gasoline consumption and 71.80% of total diesel consumption in 2007. In addition, during the study period, the energy consumption caused by the rapid development of the tertiary industry in large cities was also expanding. Using Beijing as an example, the proportion of tertiary industry’s energy consumption in Beijing increased from 19% to 41.1% from 1990 to 2008, which accounted for 49.94% of the productive energy consumption in 2008 [88].

However, the decomposition results in Table 6 indicate that the upgrading transformation of industrial structure will reduce the local PM_2.5_ concentration in the long term. Feng and Wang (2020) believe that industrial upgrading has effectively reduced the concentration of haze pollution in local cities of the eastern Chinese region [49]. Therefore, we have reason to believe that the upgrading adjustment of urban industrial structure will undergo policy throes. In the short run, the implementation of policy may greatly stimulate the development of producer services, making the upgrading transformation of industrial structure manifested to be environmentally unfriendly. Zhang et al. (2020) also found that the second industry is dominant in China, and the positive effect of industrial structure upgrading has not been shown yet [7].

**Figure 3:**
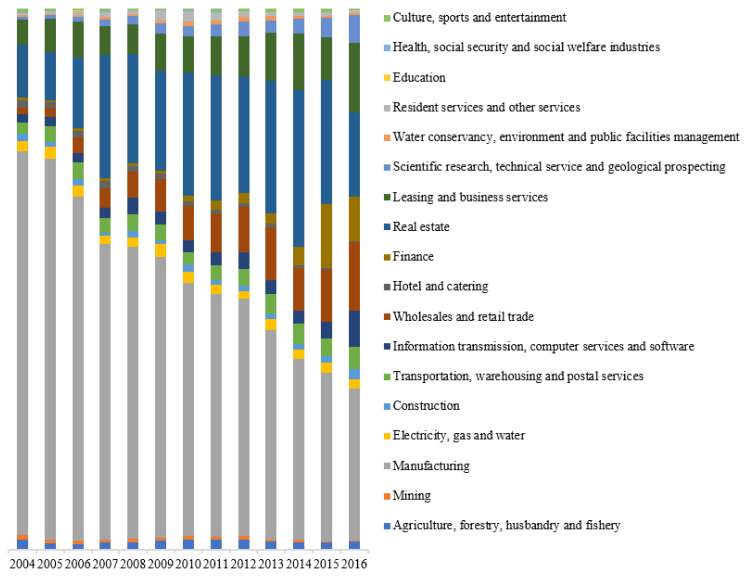
Industrial distribution of China’s FDI from 2004 to 2016.

Column (2) in Table 8 further verifies the PHH, that is, FDI entered into polluting industries mostly. Figure 3 shows the industrial distribution of China’s FDI from 2004 to 2016. It can be seen that manufacturing has been the industry with the highest proportion for many years. This indicates that the introduction of FDI has hindered the upgrading of Chinese urban industrial structure. However, due to the gradual disappearance of demographic dividend, stronger environmental regulation, and currency appreciation of China, FDI in manufacturing has also begun to decline year after year. Certain FDI in low-end manufacturing has begun to concentrate in Southeast Asian countries with lower labor costs, and FDI in high-end manufacturing has the trend of re-shoring. Based on a long-run analysis, if the structure of FDI can be continuously optimized, the impact of FDI on Chinese urban pollution will likely go from positive to negative, and pollution havens in China will also gradually decrease.

### 5.3. Labor Flux and Rationalization of Industrial Structure

In this paper, the industrial structure rationalization index mainly describes the degree of structural coupling between labor force and economic output. During the study period, both have undergone profound changes in China.

After its accession to WTO, China is embedded at the low end of the global value chain [89]. China’s economy accelerated its shift to the industrial sector, which had not yet profoundly affected labor flux. At that stage, labor flux was mainly reflected in the transfer of surplus rural labor to cities, that was, migrant workers went to cities for work and business. In 2007, Chinese industrialization further accelerated. The labor demand in large cities continued to increase. Especially in the eastern coastal cities, higher qualified labor force was required. Wang et al. (2021) showed that China has experienced significant employment polarization similar to that experienced by developed countries during that time. China’s employment polarization is significant at the industry level. The employment share of high-skilled industries represented by education, finance, and scientific research has increased significantly [89]. At this stage, the transfer of surplus rural labor began to move across cities and regions, which had become the main theme of urban labor structure transformation, but large influxes of labor to the secondary and tertiary industries exacerbated per capita output inequality between sectors. Larger structural deviations began to appear between the labor force and economy.

In Table 5 and Table 6, it can be seen that unreasonable industrial structure increased the concentration of Chinese urban PM_2.5_. This is because unreasonable industrial structure will distort the allocation of factors, increase the waste of resources, and result in inefficient development of the economy and many productive pollution emissions. Zhang et al. (2020) also holds that while optimizing the input-output structure, the coordination ability and relevant level among various industries can be improved, such as to maximize the efficiency of resource allocation and reduce environmental pollution [7]. Column (2) in Table 9 further indicates that FDI increases the irrationality of Chinese urban industrial structures, which will not help reduce PM_2.5_ pollution. This neatly illustrates that the effect of demonstration and learning-by-doing requires a reasonable allocation of factors [90]. The crude development model will not make the pollution halo a reality.

**Figure 4:**
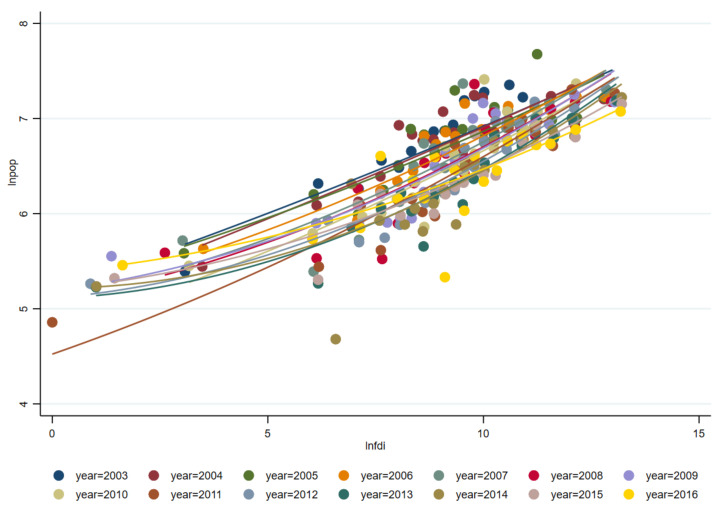
Fitting curve of population density and FDI from 2003 to 2016.

**Figure 5:**
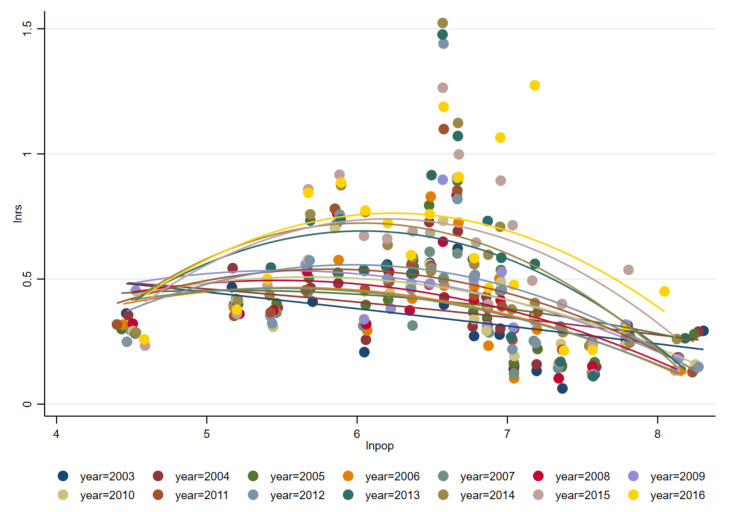
Fitting curve of industrial structure rationalization and population density from 2003 to 2016.

How does FDI affect the rationalization of Chinese urban industrial structure by promoting labor transfer? Figure 4 and Figure 5 respectively show the fitting curves of population density and FDI and industrial structure rationalization and population density from 2003 to 2016. It shows that the more FDI, the higher population density, and the higher population density, the more reasonable industrial structure.

Accordingly, it can be inferred that FDI has created more employment supply for big cities in metropolitans. Under the free movement premise of labor force, people go to big cities to find better jobs, which can help the industrial structure of big cities be rationalized [91]. However, more satellite cities break their original relatively balanced industrial structure due to the labor flux. Their industrial structure has shown a seriously unreasonable status in the short run.

Furthermore, there are large structural differences between migrant and non-migrant labor in terms of age, gender, education, etc. This has caused the satellite cities to face more structural contradictions in terms of employment and economic growth. As a whole, FDI has a negative impact on the rationalized development of Chinese urban industrial structure. It can also be seen in Figure 5 that since 2007, increasing points have jumped above the fitting line, which indicate that more Chinese cities are showing a low degree of industrial structure rationalization in the development.

## 6. Conclusions and Policy Implications

Our research focused on the effects of FDI on PM_2.5_ pollution through the lens of China’s industrial structure transformation, making it one of only a few studies to place the industrial structure at the center of this analysis. We explored a rich data set containing data on FDI inflows, industrial structure, and PM_2.5_ pollution from 2003 to 2016 for 271 cities in China. We used the STIRPAT model, a spatial econometrics method for regression analysis (Dubin, 1998), and the method proposed by Baron and Kenny (1986) to test the mediating effect of industrial structure [67,84].

Our results show that The PHH based on urban PM_2.5_ pollution is applicable in China, that is, FDI increases PM_2.5_ concentrations in Chinese cities by transferring the polluting industries. Transformation of the urban industrial structure in China will significantly affect PM_2.5_ concentrations through two paths: industrial structure upgrading and rationalization. The upgrading adjustment of urban industrial structure will undergo policy throes, but the unreasonable urban industrial structure has a continuous positive impact on PM_2.5_ concentrations. Though the mediating effect test, both have partial mediating effects on the impact of FDI on PM_2.5_. Specifically, FDI increases the PM_2.5_ concentration by hindering the industrial structure upgrading and improving the irrationality of the industrial structure.

Although our research focused on China, it has implications for other developing countries. The positive impact of FDI on PM_2.5_ indicates that FDI is both a problem and a solution for reducing urban PM_2.5_ pollution. Local governments should guide high-quality foreign investment toward industries and formulate regulations to limit the discharge of pollutants [50]. Policies should endure the throes of industrial restructuring and cannot be easily changed. In addition, it is necessary to establish a mechanism for cooperation among governments. Issues such as spatial diffusion of PM_2.5_, blind competition of FDI, labor flux between cities, and industrial layout all desperately need coordinated governance among governments. Based on the summary above, the policy implications of this paper mainly focus on the following three aspects.

First, a reasonable foreign investment management mechanism can help reduce pollution. The government should focus on promoting the market-oriented reform of FDI in the green energy-saving industry to improve the national treatment added negative list management mechanism and to continue to promote the opening progress in the green energy-saving industry. Conversely, the government should create a more convenient, legalized, and international environment for green and energy-saving foreign investment. While advancing the reform of foreign investment, government should also formulate and improve domestic foreign investment laws and regulations and relevant normative documents, with the advantage of institutional openness, to attract more high-quality and clean foreign investment into the service industry and help the green upgrade and sustainable development of China’s manufacturing.

Then, local governments should innovate the governance philosophy of PM_2.5_ and guide to optimize the industrial structure of FDI. They need to change the governance philosophy of only reducing the existing emissions and actively seek alternative energy sources, adjust the energy structure, and improve the quality of energy sources, thereby promoting the upgrading of the industrial structure and breaking the vicious circle of PM_2.5_ pollution. In addition, for manufacturing FDI, it should be guided to flow into hi-tech production capacity that meets the needs of industrial upgrading (such as unmanned vehicles, electronic chips, aerospace, biomedicine, etc.) gradually and orderly, to reduce the proportion of FDI entering into high-energy, high-polluting manufacturing. Moreover, the domestic manufacturing chain will be gradually forced to upgrade and the overall structure of FDI will be balanced and optimized.

Lastly, local governments should create top-level design of PM_2.5_ control and establish a sound market supervision system for PM_2.5_. Local governments must fully realize the “public goods” nature of PM_2.5_ control to correct for market failures. The PM_2.5_ control must be led by government intervention and supplemented by markets regulation. At the same time, local governments should also establish a sound air supervision system, strengthen the rectification and management of high-emission and high-polluting industries, and eliminate the phenomenon of unlawful discharge of pollutants.

Still, many relevant questions remain beyond our current reach. For example, since the SEDAC of Columbia University only released the PM_2.5_ data of global cities from 1998 to 2016, and China’s city-level FDI data had a large number of missing before 2002. Therefore, this study has delineated the period from 2003 to 2016, and the study period is relatively short, which may cause slight inaccuracies in the statistics results. As new data on air pollution as well as FDI are becoming increasingly available, more accurate research conclusions may be obtained in future research.

## Figures and Tables

**Table 1:** Literature list.

Author(s)	Country	Period	Methodology	Variables	Conclusion	Structural Effect
Tang and Tan (2015) [38]	Vietnam	1976–2009	The techniques of cointegration and Granger causality	CO_2_ emissions, GDP, Square of GDP, energy consumption and FDI	Pollution halo	Positive
Zhang and Zhou (2016) [28]	China	1995–2010	FE, N–W, FGLS, PCSE and DK	CO_2_ emissions, Population, GDP per capita, Technology level, industrial structure, FDI and Urbanization	Pollution halo	Negative
Zhu et al. (2016) [39]	ASEAN-5	1981–2011	Fixed effect panel quantile regression	CO_2_ emissions, Energy consumption, Economic growth, Total population, Trade openness, The industrial structure, FDI and Financial development	Pollution halo	Not significant
Jiang et al. (2016) [40]	China	2014	SLM, SEM and SDM	Air quality index, GDP per capita, Square of GDP per capita, FDI, Industrial structure and Population density	Pollution Halo	Positive
Shao et al. (2016) [41]	China	1998–2012	POLS and SGMM	PM_2.5_, population density, GDP per capita, R&D, industrial structure, energy efficiency, energy structure, road and FDI	Pollution halo	Positive
Bakirtas and Cetin (2017) [42]	MIKTA	1982–2011	PVAR estimations	CO_2_ emissions per capita, real GDP per capita, the square of real GDP per capita, the energy consumption per capita and FDI	Pollution haven	Positive
Huang et al. (2017) [43]	China	2001–2012	Spatial Durbin Model	SO_2_, FDI, GDP per square kilometer, The share of service industry in GDP, capital per worker and real income per capita	Pollution halo	Positive
Liu et al. (2017) [14]	China	2002–2015	OLS, FE and GMM	per capita SO_2_ emissions, per capita GDP, per capita fixed asset investment, per capita foreign direct investment, per capita soot emissions, ratio of secondary industry’s value-added to GDP and population density	Pollution haven	Positive
Zhu et al. (2017) [15]	China (Beijing-Tianjin-Hebei)	2000–2013	OLS and SDM	SO_2_, FDI, R&D, GDP, Population and Energy consumption	Pollution haven	Positive
Kocak and Sarkgunesi (2018) [44]	Turkey	1974–2013	Dynamic OLS	CO_2_ emissions per capita, GDP per capita, Square of GDP per Capita, Share of FDI in GDP and Energy consumption per capita	Pollution haven	Positive
Cao et al. (2018) [45]	China	2002–2013	Hierarchical linear model	SO_2_, FDI, R&D, GDP, Population and Energy consumption	Pollution halo	Positive
Salahuddin et al. (2018) [46]	Kuwait	1980–2013	ARDL and VECM Granger causality analysis	Per capita CO_2_ emissions, Per capita energy consumption, Per capita real GDP, Financial development and FDI as share of GDP	Pollution haven	Positive
Shao et al. (2019) [47]	China	1998–2013	2SLS and GS2SLS	PM_2.5_, population density, GDP per capita, technology, urbanization, industrial structure, energy structure, traffic, FDI and environmental regulation	Not significant	Positive
Wang et al. (2019) [48]	China	2015	Geographically weighted regression	PM_2.5_, Built-up area as a proportion of total area, Population density, GDP per capita, FDI, Industrial structure, Research and development, and other variables	Pollution haven	Negative
Feng and Wang (2020) [49]	China	2003–2016	Dynamic spatial Durbin model	Haze pollution, urban sprawl, per capita GDP, FDI, government intervention, electric strength, industrial upgrading, population density and environmental regulation	Pollution haven	Negative
Wang and Luo (2020) [50]	China	2006–2016	Hansen nonlinear panel threshold regression	Environmental pollution, Technological innovation capability, FDI, economic development levels, government regulation levels, industrial structures and foreign trade patterns	Pollution haven	Negative
Zhang et al. (2020) [7]	China	2006–2016	SAR and SEM	Haze pollution, Environmental regulation, Industrial structure, GDP per capita, Motor vehicles, Energy structure and FDI	Pollution haven	Positive
Amoako and Insaidoo (2021) [51]	Ghana	1981–2014	FMOLS and Canonical Cointegration Regression	Energy consumption, FDI, Financial Development, Energy Price, Trade Openness and Industry Value-Added	Pollution Heaven	Positive

**Table 2:** Variables in the empirical model.

Variables	Meaning	Mean	Std.Dev	Min	Max
PM_2.5_ [71]	PM_2.5_ (μg/m^3^)	37.39	16.21	4.52	90.86
P [75]	Population density (million people/km^2^)	999.35	970.66	12.99	14,052.41
A [75]	GDP (million yuan)	7267.53	9624.87	421.25	87,576.61
T [75]	GDP per unit of electricity (yuan/billion kwh)	1.78	1.62	0.11	16.39
FDI [75]	Foreign-invested enterprises (million yuan)	560.39	1420.81	0	25,958.26
US [75]	Industrial structure upgrading index	0.81	0.36	0.13	4.17
RS [75]	Industrial structure rationalization index	2.13	3.91	1	85.09
Light [73,74]	Stable light	9.23	9.64	0.01	60.88
PGA [75]	Park green space area (hectares)	1327.35	2558.34	1.12	30,068.99
PTR [75]	Total bus and tram traffic volume (million people)	199.22	450.29	0.01	5256.06

**Table 3:** Unit root test.

Variables	Level Value			First Difference Value		
	Rho	*Z* Value	*p* Value	Rho	*Z* Value	*p* Value
lnPM2.5	0.1965	−48.4970	0.0000	−0.3541	−85.7108	0.0000
lnP	0.6236	−14.1783	0.0000	−0.2507	−77.9361	0.0000
lnA	0.8634	5.0911	1.0000	0.2747	−38.4314	0.0000
lnT	0.6089	−15.3539	0.0000	−0.2061	−74.5819	0.0000
lnFDI	0.5666	−18.7547	0.0000	−0.2179	−75.4711	0.0000
lnUS	0.8411	3.3010	0.9995	0.0747	−53.4649	0.0000
lnRS	0.7088	−7.3265	0.0000	−0.2460	−77.5870	0.0000
lnLight	1.0069	16.6252	1.0000	0.1475	−47.9927	0.0000
lnPGA	0.6950	−8.4367	0.0000	0.1994	−44.0894	0.0000
lnPTR	0.5508	−20.0272	0.0000	−0.2052	−74.5151	0.0000

**Table 4:** Cointegration test.

Test Method	Null Hypothesis	Statistics	Statistic Value	*p* Value
Kao test	*H*_0_: *ρ* = 1	DF	−29.2898	0.0000
		ADF	−15.2352	0.0000

**Table 5:** Empirical results regarding the PHH based on PM_2.5_.

	Static Model	Dynamic Model			
	(1)	(2)	(3)	(4)	(5)
lnfdi	0.0040 **	0.0271 ***	0.3447 ***	0.0091 ***	0.2904 ***
	(0.0019)	(0.0019)	(0.0019)	(0.0019)	(0.0019)
lnus	0.0091	0.0698 ***	0.7917 ***	0.1152 ***	3.7312 ***
	(0.0113)	(0.0115)	(0.0115)	(0.0120)	(0.0121)
lnrs	−0.0393 ***	0.1193 ***	0.7649 ***	0.1311 ***	0.5331 ***
	(0.0069)	(0.0069)	(0.0069)	(0.0070)	(0.0070)
lngdp	0.1255	−4.8550 ***	40.9203 ***	−4.4281 ***	−48.3321 ***
	(0.1412)	(0.1511)	(1.5893)	(0.1557)	(1.5728)
lngdp^2^	−0.0061	0.2060 ***	−2.4813 ***	0.1777 ***	4.1616 ***
	(0.0055)	(0.0059)	(0.1210)	(0.0060)	(0.1195)
lngdp^3^			0.0423 ***		−0.1122 ***
			(0.0031)		(0.0030)
lnlight				0.1936 ***	0.0221 ***
				(0.0080)	(0.0080)
lnlight ^2^				0.0972 ***	0.3611 ***
				(0.0028)	(0.0028)
lnpop				0.1190 ***	0.2422 ***
				(0.0100)	(0.0100)
lnpga				0.0015	−0.6363 ***
				(0.0048)	(0.0048)
lneff				−0.0377 ***	−0.2705 ***
				(0.0068)	(0.0068)
lnptr				0.0299 ***	−0.3643 ***
				(0.0043)	(0.0043)
τ		0.2865 ***	−0.5209 ***	0.1393 ***	−0.6896 ***
		(0.0158)	(0.0158)	(0.0159)	(0.0159)
ρ	0.9092 ***	0.9990 ***	1.8624 ***	1.0302 ***	1.2641 ***
	(0.0214)	(0.0220)	(0.0221)	(0.0229)	(0.0229)
sigma2_e	0.0137 ***	0.0130 ***	0.0120 ***	0.0124 ***	0.0122 ***
	(0.0003)	(0.0003)	(0.0003)	(0.0003)	(0.0003)
Hausman P	0.0000				
AIC	−5434.999	−5462.607	−5461.613	−5584.739	−5581.194
BIC	−5360.105	−5382.435	−5369.107	−5430.563	−5414.683
Wald test P	0.0003	0.0000	0.0000	0.0000	0.0000
Lratio test P	0.0001	0.0000	0.0000	0.0000	0.0000
Observations	3794	3523	3523	3523	3523
Number of city	271	271	271	271	271

Notes: standard errors in parentheses; **, *** represent the significance at the 5%, and 1% level, respectively; superscript 2 and 3 respectively represent the square and cube of the variable.

**Table 6:** Decomposition of effects in the dynamic SDM model.

	SR_Direct	SR_Indirect	LR_Direct	LR_Indirect
lnfdi	0.1425	0.1620	0.0265 ***	−0.0358 ***
	(0.9617)	(1.5479)	(0.0033)	(0.0040)
lnus	−2.1435	−2.7920	−0.1220 ***	0.5740 ***
	(18.5154)	(29.7912)	(0.0365)	(0.0561)
lnrs	1.5266	1.7230	0.3108 ***	−0.3535 ***
	(11.3066)	(18.1919)	(0.0216)	(0.0340)
lngdp	−126.0700	−150.1527	−18.9900 ***	30.8987 ***
	(983.4976)	(1582.3961)	(1.8113)	(2.9719)
lngdp^2^	4.8929	5.8204	0.7432 ***	−1.1976 ***
	(38.1230)	(61.3379)	(0.0702)	(0.1151)
lnlight	−3.8612	−5.0137	−0.2354 ***	1.0266 ***
	(33.3949)	(53.7286)	(0.0625)	(0.0999)
lnlight ^2^	1.3789	1.5851	0.2577 ***	−0.3231 ***
	(10.5247)	(16.9335)	(0.0194)	(0.0311)
lnpop	0.3058	0.2318	0.1594 ***	−0.0448 ***
	(1.5613)	(2.5116)	(0.0114)	(0.0049)
lnpga	−1.2108	−1.4982	−0.1359 ***	0.3071 ***
	(9.8715)	(15.8824)	(0.0187)	(0.0300)
lneff	0.4547	0.6059	0.0130	−0.1270 ***
	(3.8013)	(6.1151)	(0.0114)	(0.0145)
lnptr	0.1306	0.1251	0.0459 ***	−0.0248 ***
	(0.8484)	(1.3645)	(0.0054)	(0.0045)

Notes: standard errors in parentheses; *** represents the significance at the 1% level; superscript 2 represents the square of the variable.

**Table 7:** Robustness test.

	(1)	(2)
lnfdi	0.0106 ***	0.0106 ***
	(0.0019)	(0.0021)
L.lnfdi		−0.0039 *
		(0.0021)
lnus	0.0753 ***	0.1157 ***
	(0.0211)	(0.0120)
lnrs	0.2969 ***	0.1296 ***
	(0.0070)	(0.0070)
lngdp	−11.5100 ***	−4.4083 ***
	(0.1523)	(0.1566)
lngdp^2^	0.4615 ***	0.1770 ***
	(0.0059)	(0.0060)
lnlight	0.4132 ***	0.1909 ***
	(0.0080)	(0.0080)
lnlight ^2^	0.2039 ***	0.0962 ***
	(0.0028)	(0.0028)
lnpop	0.2601 ***	0.1186 ***
	(0.0100)	(0.0100)
lnpga	−0.0009	0.0011
	(0.0048)	(0.0048)
lneff	−0.0744 ***	−0.0377 ***
	(0.0068)	(0.0068)
lnptr	0.0653 ***	0.0299 ***
	(0.0043)	(0.0043)
τ	−0.0905 ***	0.1404 ***
	(0.0159)	(0.0159)
ρ	1.2307 ***	1.0289 ***
	(0.0237)	(0.0230)
sigma2_e	0.0123 ***	0.0124 ***
	(0.0003)	(0.0003)
AIC	−5587.636	−5587.882
BIC	−5433.46	−5421.371
Wald test P	0.0000	0.0000
Lratio test P	0.0000	0.0000
Observations	3523	3523
Number of city	271	271

Notes: standard errors in parentheses; *, *** represent the significance at the 10% and 1% level, respectively; superscript 2 represents the square of the variable.

**Table 8:** Mediating effect of the industrial structure upgrading index.

	(1)	(2)	(3)
	lnpm_2.5_	lnus	lnpm_2.5_
lnfdi	0.0419 ***	−0.0326 ***	0.0368 ***
	(0.0093)	(0.0101)	(0.0034)
lnus			−0.1322 ***
			(0.0268)
lngdp	−19.1438 ***	−4.8730 ***	−11.2896 ***
	(3.9767)	(0.4343)	(0.8290)
lngdp^2^	0.7649 ***	0.1807 ***	0.4582 ***
	(0.1570)	(0.0174)	(0.0323)
lnlight	−0.7453 ***	−0.9503 ***	−0.2499 ***
	(0.2451)	(0.1439)	(0.0523)
lnlight^2^	0.4091 ***	0.1363 ***	0.2737 ***
	(0.0821)	(0.0267)	(0.0153)
lnpop	−0.0181	−0.0496 ***	−0.0110
	(0.0123)	(0.0186)	(0.0126)
lnpga	−0.0769 **	0.0760 ***	−0.0196 **
	(0.0342)	(0.0153)	(0.0084)
lneff	0.2556 ***	−0.3531 ***	0.0129
	(0.0819)	(0.0431)	(0.0106)
lnptr	0.0332 ***	−0.0388 **	0.0434 ***
	(0.0060)	(0.0168)	(0.0057)
L.lnpm_2.5_	0.1774 ***	−0.0981 ***	0.2501 ***
	(0.0158)	(0.0243)	(0.0158)
ρ	0.9187 ***	0.7851 ***	0.9570 ***
	(0.0228)	(0.0529)	(0.0231)
sigma2_e	0.0127 ***	0.0268 ***	0.0126 ***
	(0.0003)	(0.0006)	(0.0003)
Number of city	271	271	271

Notes: standard errors in parentheses; **, *** represent the significance at the 5%, and 1% level, respectively; superscript 2 represents the square of the variable.

**Table 9:** Mediating effect of the industrial structure rationalization index.

	(1)	(2)	(3)
	lnpm_2.5_	lnrs	lnpm_2.5_
lnfdi	0.0072 ***	0.0226 **	0.0033 *
	(0.0019)	(0.0088)	(0.0019)
lnrs			0.1591 ***
			(0.0070)
lngdp	−3.4657 ***	−2.3118 ***	−5.5445 ***
	(0.1483)	(0.5309)	(0.1491)
lngdp^2^	0.1444 ***	0.0999 ***	0.2164 ***
	(0.0058)	(0.0207)	(0.0058)
lnlight	0.1379 ***	−0.1425 **	0.1480 ***
	(0.0080)	(0.0675)	(0.0079)
lnlight ^2^	0.0831 ***	−0.0416 **	0.0849 ***
	(0.0028)	(0.0184)	(0.0028)
lnpop	−0.0152	−0.2484 ***	0.1132 ***
	(0.0099)	(0.0258)	(0.0100)
lnpga	0.0411 ***	−0.0028	0.0197 ***
	(0.0048)	(0.0159)	(0.0048)
lneff	−0.0424 ***	−0.2702 ***	−0.0541 ***
	(0.0067)	(0.0278)	(0.0067)
lnptr	0.0318 ***	−0.0296 *	0.0343 ***
	(0.0043)	(0.0171)	(0.0043)
ρ	0.9186 ***	0.1385	0.9877 ***
	(0.0228)	(0.1161)	(0.0226)
L.lnpm_2.5_	0.1769 ***	−0.2635 ***	0.0709 ***
	(0.0158)	(0.0364)	(0.0159)
sigma2_e	0.0127 ***	0.0702 ***	0.0125 ***
	(0.0003)	(0.0016)	(0.0003)
Number of city	271	271	271

Notes: standard errors in parentheses; *, **, *** represent the significance at the 10%, 5%, and 1% level, respectively; superscript 2 represents the square of the variable.

**Figure A1:**
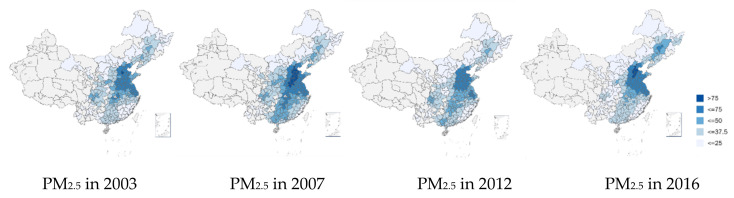
Chinese urban PM_2.5_ concentration in different years.

## Data Availability

The data presented in this study are openly available in reference number [3,12,71,73,74,75,88].

## Appendix A

When the data involves geospatial features, the observed values cannot remain independent because the closer distance may cause relevance [92]. The related paper has shown that air pollution has both dependence and heterogeneity in terms of space [68]. The dependent variable in this paper has obvious spatial conduction effects. PM_2.5_ pollution can spread and metastasize, causing the interaction effect among neighboring cities [5,66]. Thus, this paper uses Moran’s Index to test the spatial agglomeration effect of PM_2.5_. The results in Table A1 show that Moran’s Index of PM_2.5_ is positive at the 1% significance level under the inverse distance matrix, and all remaining above the level of 0.6. It indicates that the cities with high PM_2.5_ tend to be adjacent to the high PM_2.5_ cities and the low PM_2.5_ cities tend to be adjacent to the low PM_2.5_ cities. The clusters of “high-high” and “low-low” reflect a positive spatial correlation of PM_2.5_ in the 271 cities. It is necessary to consider spatial econometric models that can reflect the spatial dependence of the sample data [93].

**Table A1:** Test results of Moran’s Index for PM2.5 concentration in 271 Chinese cities.

Year	I	E(I)	sd(I)	z	*p*-Value
2003	0.6654	−0.0037	0.0185	36.0849	0.001
2004	0.6072	−0.0037	0.0193	31.6175	0.001
2005	0.6272	−0.0037	0.019	33.224	0.001
2006	0.622	−0.0037	0.0189	33.1095	0.001
2007	0.6841	−0.0037	0.0188	36.4454	0.001
2008	0.638	−0.0037	0.0191	33.6183	0.001
2009	0.6181	−0.0037	0.0189	32.9396	0.001
2010	0.6521	−0.0037	0.019	34.405	0.001
2011	0.6481	−0.0037	0.019	34.3283	0.001
2012	0.6487	−0.0037	0.0189	34.397	0.001
2013	0.6432	−0.0037	0.0187	34.5751	0.001
2014	0.6228	−0.0037	0.0189	33.0294	0.001
2015	0.6695	−0.0037	0.019	35.3273	0.001
2016	0.6604	−0.0037	0.0186	35.5898	0.001

From Figure A1, the high PM_2.5_ concentration cities in China are mainly concentrated in the Beijing-Tianjin-Hebei region and the Yangtze River Delta region. Several cities in the northeast and central China also have relatively serious PM_2.5_ pollution, but most cities in the northwest and south China are less polluted.
